# Chaotic signal denoising based on energy selection TQWT and adaptive SVD

**DOI:** 10.1038/s41598-023-45811-y

**Published:** 2023-11-01

**Authors:** Xinlu Yang, Wenbo Wang

**Affiliations:** School of Science, Wuhan University of Science and Technology, Wuhan, 430000 China

**Keywords:** Information theory and computation, Applied mathematics, Nonlinear phenomena

## Abstract

Aiming at the problem of denoising chaotic signals with low signal-to-noise ratio and unknown dynamic system parameters, a new chaotic signal denoising algorithm is proposed, which combines adjustable Q-factor wavelet transform (TQWT) and adaptive singular value decomposition (ASVD). This method uses TQWT to decompose the noisy chaotic signal. According to the maximum wavelet entropy theory and energy threshold rule, the subband of TQWT is accurately divided into signal subband and noise subband. For noise subbands, adaptive SVD is used to denoise them, to achieve preliminary denoising. In ASVD, the standard deviation of the singular value subset is used to determine the effective reconstruction order to improve the noise suppression effect. To further remove noise in the signal subband, TQWT reconstruction is performed on the preliminarily denoised signal, and ASVD is used to denoise the reconstructed signal again to obtain the chaotic signal after secondary denoising. Chua's simulated signal and four kinds of underwater radiated noise measured by TQWT-ASVD were denoised, and compared with the SVD denoising method, TQWT denoising method, complete ensemble empirical mode decomposition with adaptive noise and threshold denoising method (CEEMDAN-WT) and modified ensemble empirical mode decomposition combined with least squares denoising method (MEEMD-LMS), The experimental results show that the TQWT-ASVD method can reduce the noise of chaotic signals more effectively. Compared with SVD, TQWT, CEEMDAN-WT, MEEMD-LMS, and Chua's signal denoising method, the signal-to-noise ratio (SNR) of this method increased by 23.22%, 26.46%, 18.79%, 16.11% the root mean square error (RMSE) decreased by 32.53%,39.48%, 30.96%, 27.94%, and the row entropy (PE) decreased by 40.44%, 41.96%, 22.78%, 20.59%; After reducing the radiation noise of cargo ships, the PE value of this method is reduced by 13.91%, 10.18%, 10.88%, 8.68% respectively, and the FE value is reduced by 33.66%, 31.42%, 26.98%, 21.32% respectively.

## Introduction

Chaos is a seemingly random and irregular motion that occurs in a deterministic system^1^, preprocessing the observed data is crucial for effectively extracting the required chaotic information. However, when observing chaotic data, it is highly susceptible to noise interference, resulting in significant errors in extracting the required chaotic information. Therefore, it is necessary to perform denoising preprocessing on observed chaotic data without affecting the dynamic characteristics of the signal^2,3^.

Since the spectrum of chaotic signals often overlaps with the spectrum of noise^4^, traditional linear low-pass filtering cannot effectively denoise chaotic signals. The chaotic signal denoised by low-pass filtering will be a severely smooth transition, losing a large amount of detailed information. Therefore, how to effectively denoise chaotic signals based on their spectral characteristics is the research focus of chaotic signal-denoising.

In recent years, many chaotic signal-denoising algorithms have been proposed^3,5–17^. Singular spectrum analysis^18^ and local projection method^5,6^ are chaotic noise reduction algorithms based on phase space reconstruction. The chaotic signal and noise after phase space reconstruction have different dynamic characteristics, so this method can be used to separate chaotic signal and noise. However, the singular spectrum analysis method is difficult to accurately determine the noise boundary point for noise reduction, and the parameter selection method of local projection also has some limitations. Therefore, when the noise of a chaotic signal is serious, the noise reduction ability of the method based on phase space reconstruction will be seriously reduced. The local curve fitting algorithm performs segmented smoothing on noisy signals and uses least squares polynomial fitting to achieve signal denoising^7^. However, due to the highly nonlinear nature of chaos, it is difficult to accurately reconstruct signals using local linear approximation methods. The collaborative filtering denoising algorithm [uses the self-similarity of chaotic signals in the time domain to group similar blocks^8,9^, and the denoising effect of reconstructed signals after filtering is obvious. However, the selection of its filtering parameters is not adaptive, so the application of this method is still limited.

Threshold denoising methods are noise suppression algorithms based on time–frequency analysis, such as wavelet thresholding (WT) denoising^3^ and empirical mode decomposition (EMD) threshold denoising^10,11^. The main idea of the threshold denoising method is to first decompose the noisy signal in the time–frequency domain, and then separate the signal from the noise through threshold processing.

Hu et al. used variational modal decomposition to decompose noisy signals, selected the decomposed noise components through correlation coefficients, and then denoised the noise components using wavelet soft thresholding to obtain the denoised signal^12^. Gu et al. improved the wavelet threshold based on variational mode decomposition and calculated the optimal denoising threshold by minimizing Stein unbiased risk estimation, achieving good denoising results^13^. Liu et al. used wavelet packets to decompose chaotic signals and then used fuzzy analysis to construct wavelet packets the denoising thresholds to achieve the denoising of chaotic signals^14^.

Huang et al. utilized EMD to decompose the signal and then constructed an adaptive soft threshold algorithm to filter and process the various frequency band components after EMD decomposition^15^. Chen et al. combined the improved IENEMD algorithm with the adaptive threshold (ATD) for signal denoising, where ATD is calculated based on the Pearson correlation coefficient and semi-soft threshold, which has better adaptability^16^. Yang et al. combined Complete Ensemble Empirical Mode Decomposition with Adaptive Noise Analysis (CEEMDAN) with wavelet packet thresholding. They first used the correlation coefficient method to select the noise components after CEEMDAN decomposition and then used the wavelet packet thresholding to denoise the high-frequency noise components. This method improves the disadvantage of EMD denoising methods that may lose useful components^17^.

However, the denoising performance of the wavelet threshold method is greatly affected by the wavelet basis and the number of decomposition layers, which reduces the adaptability of the method. Although the EMD threshold denoising method overcomes the problem of selecting basis functions, the lack of a theoretical basis makes it very difficult to determine a more suitable threshold. Tunable Q-factor wavelet transform (TQWT) is a new type of discrete wavelet transform proposed by Selenick in 2011^19^. TWQT can freely and flexibly construct wavelet basis functions by adjusting its quality factor Q and redundancy factor r, adaptive optimal matching of wavelet basis functions and decomposition layers can be achieved based on the characteristics of the signal, thus overcoming the difficulty of selecting wavelet bases and decomposition layers in traditional wavelet transform denoising. TQWT can more effectively remove noise, but due to the overlapping spectrum of chaotic signals and noise, a single TQWT method is difficult to completely remove noise from chaotic signals.

Given this, this article proposes a chaotic signal denoising method that combines TQWT with adaptive SVD. This method first utilizes TQWT to decompose chaotic signals and determines the noise frequency band based on the frequency band energy ratio and relative wavelet entropy. Using ASVD to process the noise frequency band to achieve preliminary denoising of chaotic signals, and using the standard deviation of singular value subsets in ASVD to determine the effective reconstruction order. Then, the preliminary denoised signal is reconstructed using TQWT, and the reconstructed signal is subjected to secondary denoising using ASVD to obtain the final denoised chaotic signal. Finally, the effectiveness of the proposed method was verified by simulating chaotic signals and measuring chaotic signals.

The main structure of this article is as follows. In Section “The principle of tunable Q-factor wavelet transform (TQWT)”, the basic theories of TQWT and SVD are introduced. In Section “TQWT noise frequency band selection based on energy proportion”, a preliminary denoising method based on TQWT and a secondary denoising method based on adaptive SVD are presented. In Section “SVD denoising based on singular value subset order determination”, the basic steps of the proposed method are presented. In Section “Experiment and analysis”, noise reduction experiments are conducted using Lorenz chaotic signals and measured chaotic signals, and the proposed method is compared and analyzed with the other four methods for noise reduction. The conclusion will be drawn in Section “Conclusion”.

## The principle of tunable Q-factor wavelet transform (TQWT)

The TQWT method was originally proposed in 2011 as an improved time–frequency analysis method based on the oscillation characteristics of wavelet basis. The main adjustable parameters of TQWT are Q-factor, oversampling rate (redundancy) $r$, and decomposition level $J$. The Q-factor is defined as

1 $$Q=\frac{f}{B}$$

where the Q-factor is obtained by the ratio of the signal center frequency $f$ to the signal bandwidth $B$. The larger the value of the Q-factor, the higher the vibration attribute of the corresponding wavelet basis function. The redundancy coefficient is obtained by the ratio of the total number of wavelet coefficients to the signal length, which can be used to explain how much spectral overlap exists between adjacent bandpass filters, usually using $r$ = 3. The number of decomposition layers $J$ represents both the number of stages (levels) in TQWT and the number of filter banks. TQWT decomposition and reconstruction are composed of J-layer filter banks, totaling $J$ + 1 subbands.

By adjusting these three parameters, the scaling factor of the filter bank can be changed $\alpha$ and $\beta$. Thus, a wavelet basis that fits the frequency characteristics of the signal is obtained, and the relationship between the parameters of TQWT is as follows:

2 $$\left\{\begin{array}{l}\alpha =1-\frac{2}{\left(Q+1\right)r}\\ \beta =\frac{2}{Q+1} \end{array}\right.$$

**Figure 1:**
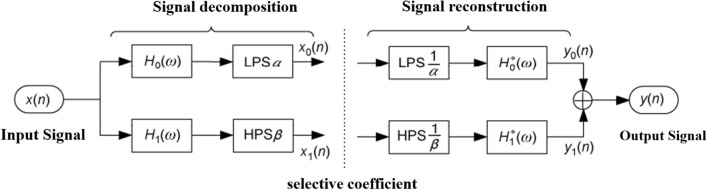
Block diagram of TQWT analysis and synthesis filter bank.

TQWT adopts a reversible oversampling filter bank with a real value sampling factor to realize signal decomposition and reconstruction. Its structure is shown in Fig. 1, where LPS and HPS represent low-pass scaling and high-pass scaling, respectively. $\alpha$ and $\beta$ Represent low-pass scaling and high-pass scaling parameters, respectively.

**Figure 2:**
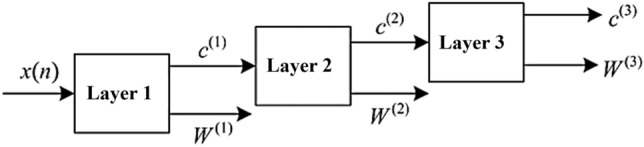
Three-layer TQWT decomposition process ($J$ = 3).

The TQWT wavelet uses the above two-channel filter banks to iterate the low-pass channels of the signal, decomposing the signal into $J$ + 1 narrowband signals. The decomposition process of the three-layer TQWT is shown in Fig. 2. To achieve perfect reconstruction, the frequency response ${H}_{i}(\omega )$, $i$ = 0, 1 of TQWT should meet the following requirements:

3 $${H}_{0}^{2}\left(\omega \right)+{H}_{1}^{2}\left(\omega \right)=1$$

Among them, ${H}_{0}\left(\omega \right)\mathrm{ and }{H}_{1}(\omega )$ defined as:

4 $${H}_{0}\left(\omega \right)=\left\{\begin{array}{l}1, \\ \theta (\frac{\omega +\left(\beta -1\right)\pi }{\alpha +\beta -1}), \\ 0, \end{array}\right.\begin{array}{c}\left|\omega \right|\le \left(1-\beta \right)\pi ,\\ \left(1-\beta \right)\pi \le \left|\omega \right|\le \alpha \pi ,\\ \alpha \pi \le \left|\omega \right|\le \pi ,\end{array}$$

5 $${H}_{1}\left(\omega \right)=\left\{\begin{array}{l}0, \\ \theta (\frac{\alpha \pi -\omega }{\alpha +\beta -1}), \\ 1, \end{array}\right.\begin{array}{c}\left|\omega \right|\le \left(1-\beta \right)\pi ,\\ \left(1-\beta \right)\pi \le \left|\omega \right|\le \alpha \pi ,\\ \alpha \pi \le \left|\omega \right|\le \pi ,\end{array}$$

The values of scaling parameters $\alpha$ and $\beta$ meet $0<\beta \le 1$,$0<\alpha <1$,$\alpha +\beta >1$. The definition of function $\theta (\omega )$ is:

6 $$\theta \left(\omega \right)=1/2(1+cos\omega )\sqrt{2-cos\omega } , |\omega |\le \pi .$$

Considering the bandwidth limitation of the filter, the maximum decomposition level of TQWT is usually determined by the following equation:

7 $${J}_{max}=\left \lfloor \frac{\mathit{lg}(\beta N/8)}{\mathit{lg}(1/\alpha )}\right \rfloor$$

where $N$ represents the length of the signal.

## TQWT noise frequency band selection based on energy proportion

Compared to traditional wavelet analysis methods, the tunable Q-factor wavelet transform can set the value of its Q-factor according to the changes in waveform characteristics without relying on wavelet basis functions. Assuming that the noisy mixed signal is:

8 $$y\left(k\right)=x\left(k\right)+n\left(k\right)$$

In the above equation, $x(k)$ is the original signal, and $n(k)$ is the superimposed Gaussian white noise signal. After setting the $Q$ factor and redundancy factor $r$ of TQWT, the decomposition level $J$ can be obtained through Eq. (7). After TQWT decomposition, $J$ + 1 subbands can be obtained.

### Frequency band energy ratio of TQWT

TQWT can concentrate the energy of useful signals on a few wavelet coefficients, while white noise is still white noise after TQWT decomposition. According to wavelet decomposition theory, the wavelet coefficients of useful signals are usually larger than those of noise with scattered energy and smaller amplitude. Therefore, after the TQWT decomposition of chaotic signals containing noise, the energy of the noise components is mainly concentrated in the high-frequency subbands and distributed evenly, while the energy of chaotic signals is concentrated in a few wavelet subbands with larger amplitudes. According to the distribution characteristics of signal and noise wavelet coefficients, a suitable subband selection threshold can be selected to truncate the wavelet subband. Zero the wavelet subbands below this threshold, preserve the wavelet subbands above this threshold, and then perform the inverse transformation on the retained TQWT subband coefficients to obtain the denoised chaotic signal.

However, the setting of the subband selection threshold is very important, which directly affects the denoising effect of TQWT. This article is based on the energy ratio of noise and signal and optimizes the subband selection threshold of TQWT through the frequency band energy ratio. Assuming that the subband coefficient after TQWT decomposition is ${w}^{j}\left(k\right)$,$j$ = 1, 2, …, J + 1, $k$ = 1, 2, …, $N$, the energy of the $j$ th subband is defined as:

9 $${E}^{j}={\sum }_{k=1}^{N}|{w}^{j}(k){|}^{2}$$

The total energy of the frequency band is defined as:

10 $$E={\sum }_{j=1}^{J+1}{E}^{j}$$

Calculate the percentage of each subband energy ${E}^{j}$ in the total energy $E$ of the frequency band:

11 $${P}^{j}=\frac{{E}^{j}}{E}$$

${P}^{j}$ is called the percentage of subband energy. Sort the subband signals according to the percentage of energy in the subband, and the sorted result can be assumed to be:

12 $$\{[{\widetilde{P}}^{1},{\widetilde{P}}^{2},\ldots ,{\widetilde{P}}^{J},{\widetilde{P}}^{J+1}]), \, \mathrm{ where } \, {\widetilde{P}}^{j}>{\widetilde{P}}^{j+1}, \,\,\, 1\le j\le J$$

Calculate the cumulative energy ratio of each subband:

13 $$C{P}^{j}={\sum }_{m=1}^{j}{\widetilde{P}}^{m}$$

Set cumulative energy ratio threshold $\lambda$, Accumulate the proportion of subband energy according to Eq. (13) until it exceeds the threshold of cumulative energy ratio $\lambda$. When stopping, the effective subband value $[{\widetilde{P}}^{1}, {\widetilde{P}}^{2},\ldots ,{ \widetilde{P}}^{j}]$ during denoising can be determined.

### Adaptive determination of cumulative energy ratio threshold

In the process of frequency band selection based on energy ratio in TQWT, the selection of the cumulative energy ratio threshold $\lambda$ is crucial for effectively separating signals from noise. If the cumulative energy ratio threshold is too small, the frequency band containing valid information will be missed. If the cumulative energy ratio threshold is too large, it will still result in a large amount of noise in the reconstructed signal. This article aims to adaptively determine the cumulative energy ratio threshold through noise variance and signal energy $\lambda$.

According to the noise variance estimation formula proposed by Donoho, the noise standard deviation of the noisy signal after wavelet decomposition is:

14 $${\sigma }_{n}=\frac{median(|{w}^{1}(k)|)}{0.6745},1\le k\le N$$

where $median\{\cdot \}$ represents the median value, ${W}^{(1)}$ represents the first layer of high-frequency wavelet coefficients after wavelet decomposition of the signal, and $n$ represents the length of the signal. According to Eq. (9), the energy mean of noisy signals can be expressed as:

15 $${\varepsilon }_{y}^{2}=\frac{1}{N}{\sum }_{k=1}^{N}{y}^{2}(k)$$

The ratio of the variance of noise energy ${\sigma }_{n}^{2}$ to the mean of signal energy ${\varepsilon }_{y}^{2}$ can approximately represent the energy occupied by noise in noisy signals. Therefore, the threshold for the cumulative energy ratio can be set as:

16 $$\lambda =1-\frac{{\sigma }_{n}^{2}}{{\varepsilon }_{y}^{2}}$$

When the cumulative energy ratio of the first ${j}_{0}$ subbands satisfies the following equation. It is considered that the selected subband already includes all signal subbands, and the remaining subbands $[{\widetilde{P}}^{{j}_{0}+1},\ldots ,{ \widetilde{P}}^{J+1}]$ are noise subbands.

17 $$C{P}^{{j}_{0}}={\sum }_{m=1}^{{j}_{0}}{\widetilde{P}}^{m}\ge \lambda$$

**Figure 3:**
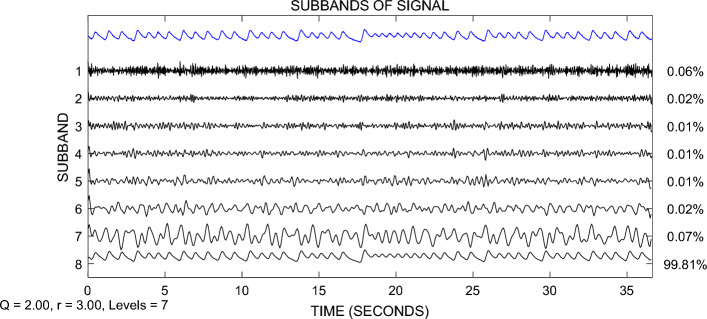
Schematic diagram of TQWT decomposition.

**Figure 4:**
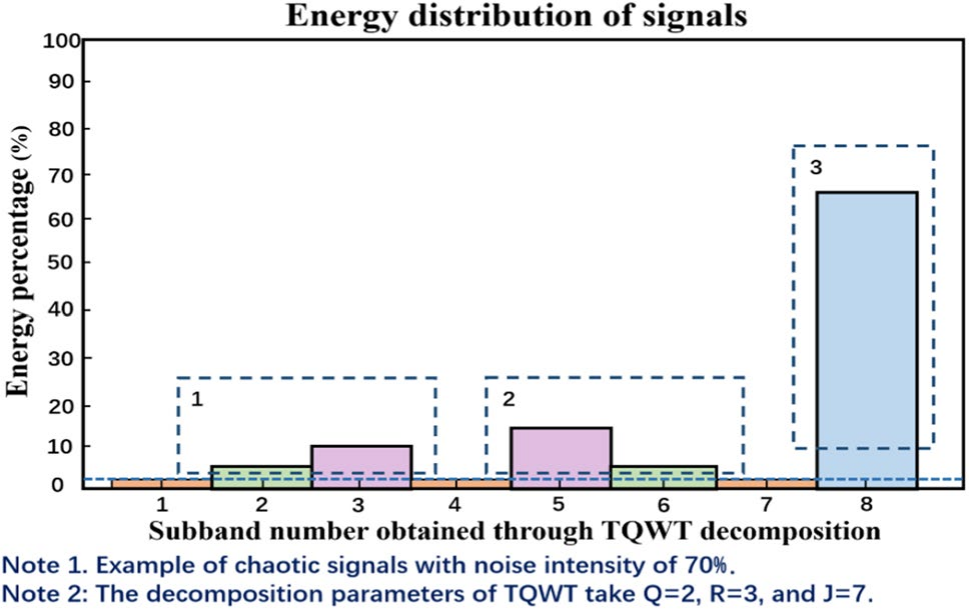
Schematic diagram of subband energy proportion.

The Lorenz system is used to generate a chaotic signal with a length of $N$ = 1024, and white noise with an SNR of 70% is added to it. TQWT is used to decompose the signal, Q-factor $\mathrm{Q}$ = 2, redundancy factor $r$ = 3, determine the number of decomposition layers $J$ = 7, and finally, 8 subbands are obtained, as shown in Fig. 3, and the subband energy distribution is shown in Fig. 4. In Fig. 3, the first line shows the Lorenz signal polluted by noise, and the second to ninth lines show the TQWT decomposition results (${W}^{(1)},{W}^{(1)},\ldots ,{W}^{(8)}$). It can be seen that the vast majority of noise is concentrated in the wavelet coefficients ${W}^{(1)}$ of the first layer, and ${W}^{(1)}$ is basically composed of noise. From Fig. 4, it can be seen that the subband energy after TQWT decomposition varies greatly. The blue dashed line in Fig. 4 represents the energy percentage threshold, and the subbands in dashed boxes 1, 2, and 3 are above a certain energy percentage ratio. The specific subbands that contain most of the energy are generally considered signal subbands, such as subbands 3, 5, and 8 in Fig. 4. These three subbands concentrate the majority of the signal's energy and can therefore be identified as signal subbands. The energy of the 1st, 4th, and 7th subbands is very small and can be considered noise subbands based on their energy ratio.

## SVD denoising based on singular value subset order determination

After selecting the signal sub-bands $[{\widetilde{P}}^{1},{ \widetilde{P}}^{2},\ldots ,{ \widetilde{P}}^{{k}_{0}}]$ and noise sub-bands $[{\widetilde{P}}^{{k}_{0}+1},{ \widetilde{P}}^{{k}_{0}+2},\ldots ,{ \widetilde{P}}^{K}]$, the signal sub-bands $[{\widetilde{P}}^{1},{ \widetilde{P}}^{2},\ldots , { \widetilde{P}}^{{k}_{0}}]$ can be directly used for TQWT inverse transformation to obtain the denoised chaotic signal. However, due to the presence of some signal details in the noise subband and a certain amount of noise in the signal subband, directly using the signal subband reconstruction to obtain denoised signals will to some extent affect the denoising effect of chaotic signals. To further preserve the detailed information of the signal and remove noise as much as possible, this paper adopts an improved singular value decomposition (SVD) method to perform secondary denoising on the noise and signal subbands.

### SVD noise reduction principle

Assuming that a noise subband signal selected by TQWT is $\mathbf{w}=[{\mathrm{w}}_{1},{\mathrm{w}}_{2},\ldots ,{\mathrm{w}}_{\mathrm{N}}]$, according to the phase space reconstruction theory of chaotic signals, the Hankel matrix of $\mathbf{w}$ can be constructed as follows:

18 $${H}_{p\times q}=\left[\begin{array}{cccc}{w}_{1}& {w}_{2}& \ldots & {w}_{q}\\ {w}_{2}& {w}_{3}& \ldots & {w}_{q+1}\\ \ldots & \ldots & \ldots & \ldots \\ {w}_{p}& {w}_{p+1}& \ldots & {w}_{N}\end{array}\right]$$

Among them, $N$ represents the length of the subband signal, $N=p+q-1$, and $p\ge q$. By performing SVD decomposition on $H$, the following equation can be obtained:

19 $$H=U\Sigma {V}^{T}$$

where $U$ and ${V}^{T}$ are matrices of sizes $p\times p$ and $q\times q$, respectively. $\Sigma$ is a diagonal matrix of size $p\times q$, with the main diagonal element ${\lambda }_{i}$ ($i=\mathrm{1,2},\ldots ,k$), i.e.:

20 $$\Sigma =diag\left({\lambda }_{1},{\lambda }_{2},\ldots ,{\lambda }_{k}\right)$$

where, ${\lambda }_{1},{\lambda }_{2},\ldots ,{\lambda }_{k}$ represents the singular value of matrix $H$, and ${\lambda }_{1}\ge {\lambda }_{2}\ge \ldots \ge {\lambda }_{k}\ge 0$,$k=\mathit{min}(p,q)$.

According to the theory of singular value decomposition and the best approximation theorem of matrices^20–24^, it is known that after the SVD decomposition of noisy signals, the singular values corresponding to the real signal are relatively large, mainly reflected by the first $r$ larger singular values. The singular values corresponding to noise are often small, mainly reflected by the last $k$-$r$ singular values. And the singular values representing the signal and noise will undergo a sudden change at a certain singular value point. If the singular value mutation point is used as the boundary point between the signal and noise, the first $r$ singular values representing the signal are retained, and the remaining $k$–$r$ singular values representing the noise are set to 0. By reconstructing the signal based on the SVD inverse transformation, the denoised signal can be obtained. That is to say, after determining the first $r$ singular values representing the signal, the Hankel matrix can be rewritten as:

21 $$H=U\Sigma {V}^{T}=U\left[\begin{array}{cc}{\Sigma }_{r}& 0\\ 0& {\Sigma }_{k-r}\end{array}\right]{V}^{T}$$

After setting $k$-$r$ singular values representing noise to 0, the estimation matrix of $H$ can be obtained as follows:

22 $$\widehat{H}=U\left[\begin{array}{cc}{\Sigma }_{r}& 0\\ 0& 0\end{array}\right]{V}^{T}$$

Matrix $\widehat{H}$ is the best approximation matrix with rank r of $H$. In matrix $\widehat{H}$, noise has been greatly compressed. By adding and averaging the anti-diagonal elements of $\widehat{H}$, the denoised signal can be obtained^20–24^. From this, it can be seen that the key to the SVD denoising algorithm is how to determine the order $r$ of the effective rank of the Hankel matrix and achieve accurate division of the signal space and noise space.

### Determination of effective rank order based on singular value adaptive grouping

The essence of SVD decomposition denoising is to group singular values based on their size relationship, that is, to find the mutation points of singular values and determine the order of the effective rank of the Hankel matrix. At present, methods for determining the effective rank order include the Singular value percentage method^21^, singular spectral analysis^22^, the relative rate of change method^23^, truncated SVD^24^, etc. However, the values of existing methods all have a certain degree of subjectivity. To determine the effective rank order more accurately and objectively, this paper proposes a method based on singular value adaptive subsets to determine the order $r$ of the effective rank. This method can automatically find the optimal boundary point for singular values, and the specific steps are as follows:

- Step 1. Arrange the singular values in ascending order. Let $A{r}_{1}={r}_{k},A{r}_{2}={r}_{k-1},\ldots ,A{r}_{k-1}={r}_{2},A{r}_{k}={r}_{1}$, and the sorted singular value is:
  23 $$\left[A{r}_{1},A{r}_{2},\ldots ,A{r}_{k-1},A{r}_{k}\right], A{r}_{1}\le A{r}_{2}\le \cdots \le A{r}_{k-1}\le A{r}_{k}$$
- Step 2. Create a singular value subset, which is composed of singular value 1 to the singular value $i$, i.e.:
  24 $${{S}_{1}=\left[A{r}_{1}\right],S}_{2}=\left[A{r}_{1},A{r}_{2}\right],\ldots {S}_{i}=\left[A{r}_{1},A{r}_{2},\ldots A{r}_{i}\right], \ldots {S}_{k}=\left[A{r}_{1},A{r}_{2},\ldots A{r}_{k}\right]$$
- Step 3. Calculate the standard deviation for each subset:
  25 $${\sigma }_{i}=\sqrt{\frac{1}{i}{\sum }_{s=1}^{i}(A{r}_{s}-\overline{A{r}_{i}}}{)}^{2}, A{r}_{s}\in {S}_{i},i=\mathrm{1,2},\ldots ,k$$
- Step 4. Calculate the integer lower bound ${\Delta }_{i}$ of the standard deviation for each subset ${\sigma }_{i}$:
  26 $$\Delta \left(\mathrm{i}\right)=\left \lfloor |{\upsigma }_{\mathrm{i}}\right |$$
  $\left \lfloor{\upsigma }_{\mathrm{i}}\right \rfloor$ represents the maximum integer that is not greater than ${\sigma }_{i}$.
- Step 5. Adaptive search for mutation point positions in singular value subsets. When $\Delta ({i}_{0})>0$ and $\Delta ({i}_{0}-1)=0$ appear for the first time, denote ${i}_{0}$ as the mutation point of the singular value subset.
- Step 6. Updating Singular Values of Hankel Matrices, $${r}_{i}{\prime}=\left\{\begin{array}{c}{r}_{i}, i\ge {i}_{0}\\ 0, i<{i}_{0}\end{array}\right.$$, Reconstruct using the updated singular values $[{r}_{i}{\prime}{]}_{i=\mathrm{1,2},\ldots ,k}$ to obtain the denoised signal.

**Figure 5:**
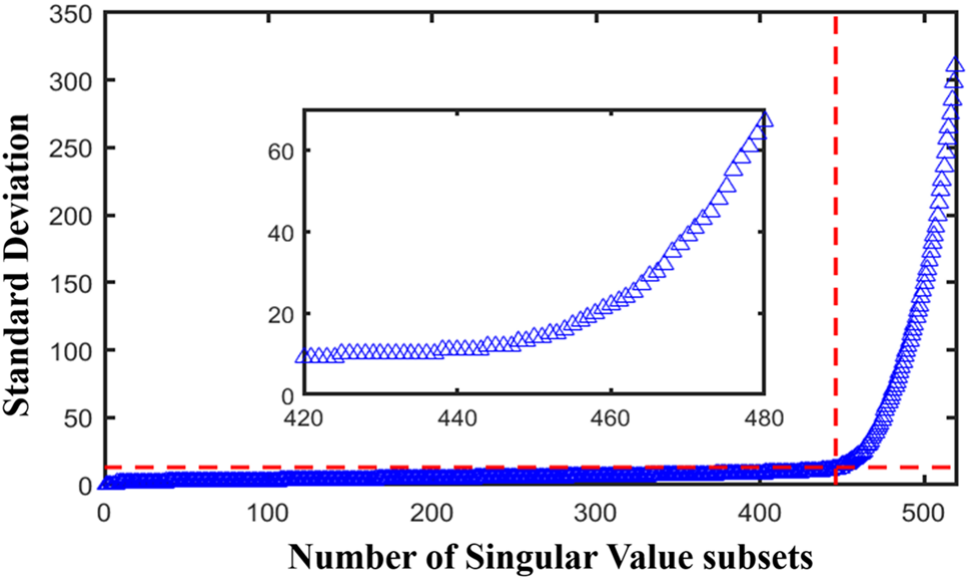
Standard deviation values of singular value subsets.

Utilize the Lorenz system to generate a chaotic signal with a length of $N$=1024 and a noise intensity of 70% for singular value decomposition, and arrange the obtained singular values in descending order. Calculate the standard deviation of the singular value subset according to Eq. (23)–(26), as shown in Fig. 5. A local magnification of Fig. 5 clearly shows that $i$=438 is the mutation point of the singular value subset. The slope of the singular value standard deviation change before the mutation point is relatively small, and the slope of the singular value standard deviation change after the mutation point is very large.

### Joint TQWT and adaptive SVD noise reduction algorithm process

The chaotic signal contaminated by noise is decomposed by TQWT, and noise and signal subbands are selected through the proportion of subband energy. For noise subbands, use the adaptive SVD algorithm in Section “Determination of effective rank order based on singular value adaptive grouping” for noise reduction processing. Perform TQWT inverse transformation on the denoised noise and signal subbands to obtain the preliminary denoised chaotic signal. To enhance the smoothness of the signal and further remove noise from the signal, an adaptive SVD decomposition is used to perform secondary noise reduction on the reconstructed chaotic signal.

The specific steps for joint denoising of TQWT and improved SVD (TQWT-adaptive SVD, TQWT-ASVD) are as follows:

1. Perform TQWT decomposition on chaotic signals to obtain wavelet coefficients $\{{{\varvec{w}}}^{j}{\}}_{j=\mathrm{1,2},\ldots , J}$ corresponding to the number of decomposed subbands. Sort the subband signals according to the percentage of energy in the energy subband, and assume that the sorted result is $\{{\widetilde{{\varvec{w}}}}^{j}{\}}_{j=\mathrm{1,2},\ldots , J}$.
2. Calculate the cumulative energy ratio threshold $\lambda$ according to Eq. (16), and filter the signal sub-band $[{\widetilde{{\varvec{w}}}}^{1},{\widetilde{{\varvec{w}}}}^{2},\ldots ,{\widetilde{{\varvec{w}}}}^{{j}_{0}}]$ and noise sub-band $[{\widetilde{{\varvec{w}}}}^{{j}_{0}+1},{\widetilde{{\varvec{w}}}}^{{j}_{0}+2},\ldots ,{\widetilde{{\varvec{w}}}}^{J}]$ through Eq. (17).
3. For the noise sub-band $[{\widetilde{{\varvec{w}}}}^{{j}_{0}+1},{\widetilde{{\varvec{w}}}}^{{j}_{0}+2},\ldots ,{\widetilde{{\varvec{w}}}}^{J}]$, use the improved SVD algorithm in Section “Determination of effective rank order based on singular value adaptive grouping” for noise reduction, and set the processed noise sub-band as $[{\widetilde{{\varvec{w}}}}_{d}^{{j}_{0}+1},{\widetilde{{\varvec{w}}}}_{d}^{{j}_{0}+2},\ldots ,{\widetilde{{\varvec{w}}}}_{d}^{J}]$.
4. The signal sub-band $[{\widetilde{{\varvec{w}}}}^{1},{\widetilde{{\varvec{w}}}}^{2},\ldots ,{\widetilde{{\varvec{w}}}}^{{j}_{0}}]$ and the noise sub-band $[{\widetilde{{\varvec{w}}}}_{d}^{{j}_{0}+1},{\widetilde{{\varvec{w}}}}_{d}^{{j}_{0}+2},\ldots ,{\widetilde{{\varvec{w}}}}_{d}^{J}]$ processed by SVD are used as inputs for TQWT reconstruction to obtain the first denoised chaotic signal.
5. Using ASVD to perform secondary denoising on the signal after the first denoising, enhancing smoothness, and obtaining the final denoised chaotic signal.

**Figure 6:**
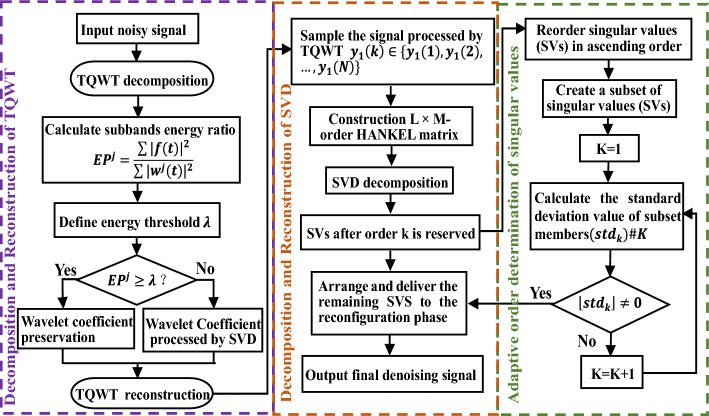
Flow chart of the improved algorithm in this article.

Based on the above analysis, the process of chaotic signal denoising algorithm based on TQWT-ASVD is shown in Fig. 6.

## Experiment and analysis

This article will conduct a denoising experimental analysis on the chaotic signals of the classic Chua's circuit and the underwater radiated noise signals of ships to verify the effectiveness of the proposed TQWT-ASVD secondary denoising method. The experimental environment is Windows 10, the CPU is Core i7-7500, the memory is 8 GB, and the compilation and running environment is Matlab-2020a. To compare the noise reduction effect of the proposed method, fitting error minimum SVD (FEM-SVD)^25^, TQWT^26^, CEEMDAN combined with threshold (CEEMDAN-WT)^27^, MEEMD combined with least mean square (MEEMD-LMS)^28^, and the proposed method (TQWT-ASVD) for denoising analysis.

### Evaluation indicators for noise reduction effect

To compare the denoising effects of different algorithms, this article uses three indicators: signal-to-noise ratio (SNR), root mean square error (RMSE), and normalized permutation entropy (PE) to measure the denoising effect of chaotic signals. The formulas for signal-to-noise ratio (SNR) and root mean square error (RMSE) are as follows:

27 $$SNR=10\times {log}_{10}\left[\frac{var\left(\widehat{s}\left(n\right)\right)}{var\left(\widehat{s}\left(n\right)-s(n)\right)}\right]$$

28 $$RMSE=\sqrt{{\sum }_{n=1}^{N}{(\widehat{s}\left(n\right)-s(n))}^{2}/N}$$

where $s(n)$ represents the real signal, $\widehat{s}\left(n\right)$ represents the denoised signal, N represents the data length, and $\mathit{var}(\cdot )$ represents the variance. The SNR reflects the denoising ability of the algorithm, and the larger its value, the better the denoising effect. RMSE reflects the average deviation between the denoised signal and the original signal, and the smaller its value, the better the denoising effect.

There are a total of $m!$ possible sorting indices for $m$-dimensional sequences. The type of sorting index that occurs is denoted as $J$, and the probability of the occurrence of the $j$th sorting index is defined as ${p}_{j}$, $1\le j\le J$, then the formula for calculating the normalized sorting entropy is:

29 $${H}_{PE}\left(m\right)=\frac{-{\sum }_{j=1}^{J}{p}_{j}{\mathit{log}}_{2}{p}_{j}}{{\mathit{log}}_{2}m!}$$

For the time series {${X}_{i}$,$i$=1, 2, …, n}, taking $m$ as the window, the time series is divided into $k$ = $n$ – $m$ + $1$ series. Calculate the Chebyshev distance ${d}_{ij}$ between each sequence and all $k$ sequences, calculate the fuzzy membership ${D}_{ij}^{m}$ from the distance ${d}_{ij}$, and average ${\Phi }^{m}(t)$ for all membership degrees except itself. Then the window length is increased to $m$ + 1, and ${\Phi }^{m+1}(t)$ is obtained. The formula for calculating fuzzy entropy is:

30 $${H}_{FE}\left(m\right)=ln{\Phi }^{m}\left(t\right)-ln{\Phi }^{m+1}\left(t\right)$$

${H}_{PE}$ and ${H}_{FE}$ can effectively measure the complexity of time series and detect dynamic changes in time series, so they can be used to quantify the complexity of denoised signals. The smaller the ${H}_{PE}$ and ${H}_{FE}$ value, the more regular the data after denoising, and the better the denoising effect.

### Chaotic signal noise reduction of Chua's circuit

Chua's circuit is the first physical achievement achieved by researchers in the field of chaos. The normalization equation of its system is as follows.

31 $$\left\{\begin{array}{l}\dot{x}=\alpha [y-x-f(x)]\\ \dot{y}=x-y-z \\ \dot{z}=-\beta y \end{array}\right.$$

32 $$f\left(x\right)=bx+\left(a-b\right)\left(\left|x+1\right|-\left|x-1\right|\right)/2$$

**Figure 7:**
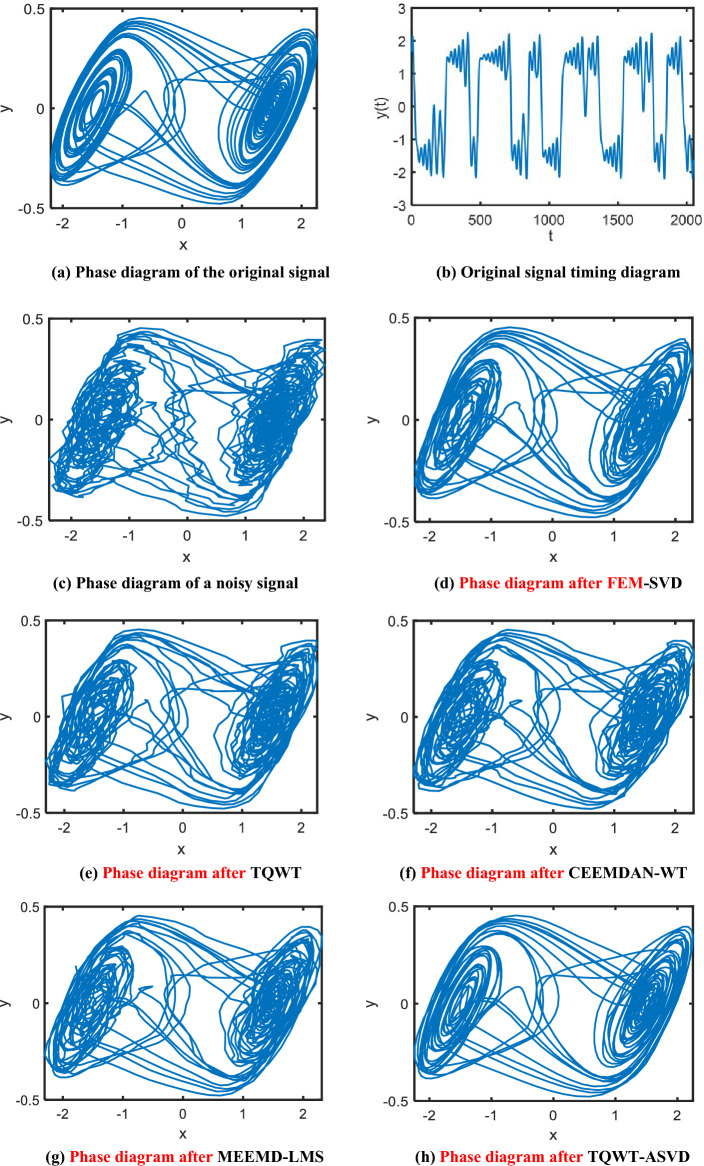
Comparison of the phase diagram of simulated Chua's chaotic signal after noise adding (SNR = 25) and denoising by FEM-SVD, TQWT, CEEMDAN-WT, MEEMD-LMS, and TQWT-ASVD methods.

Analyze the equilibrium point and Lyapunov exponent of a differential Eq. (32). When the parameters are $\alpha$=10, $\beta$=15.68, $a$=  − 1.276, $b$=  − 0.6888, the Chua circuit is in a chaotic state, and the two-vortex chaotic attractor appears. Taking the initial value [0.1, 0.1, 0.1], the fourth-order Runge–Kutta algorithm was used to simulate the interval [0, 300], and 5, 589 × 3 data were generated in total. The last 2^11^ × 3 sampling points were taken as experimental data, and the sampling frequency was 55.89 HZ. Gaussian white noise with an SNR of 25 dB was added to the simulation signal, and then the FEM-SVD, TQWT, CEEMDAN-WT, MEEMD-LMS and the method proposed in this paper were respectively used for denoising processing. The two-dimensional phase space trajectory diagram of the original signal, the denoised signal, and the five de-denoised signals are shown in Fig. 7.

Figure 7a and b respectively show the phase space and time sequence diagram of the original Chua's circuit signal, and Fig. 7c–h respectively show the phase space diagram of the signal with noise, the FEM-SVD, the TQWT, the CEEMDAN-WT, the MEEMD-LMS denoising algorithms and the signal denoised by the method in this paper.

It can be seen from Fig. 7c that the phase space of the chaotic signal of Chua's circuit after adding noise is in a very chaotic state. From Fig. 7e–g, it can be seen that after noise reduction using the TQWT, CEEMDAN-WT, and MEEMD-LMS methods, the high-frequency noise in the signal is well removed, the interference of noise on Phase space is suppressed to a certain extent, and the Phase space epidemic curve becomes relatively smooth. However, compared with the Phase space of the original Chua's circuit chaotic signal, it can be seen that there is still a certain amount of noise left in the signal after noise reduction, resulting in no obvious repair effect on the overall popular structure of the Phase space As can be seen from Fig. 7d, after the chaotic signal of Chua's circuit is de-noised by FEM-SVD algorithm, the overall popular structure of the signal Phase space has been better repaired, and the Phase space curve of the signal is smoother after noise reduction. However, it can be seen that some curves in the Phase space are not smooth enough, the regularity of the fluid–structure in the Phase space has also been damaged to a certain extent, and some fluid structures still appear messy. It can be seen from Fig. 7h that after the TQWT-ASVD method proposed in this paper for noise reduction, the overall manifold structure of the phase space and the smoothness of the manifold curve have been well repaired. The phase space track of the signal after noise reduction is smoother and more regular, and the similarity is significantly higher than the phase space of the original Chua's circuit chaotic signal. Therefore, it can be seen from Fig. 7 that the TQWT-ASVD method proposed in this paper can better remove the noise in the chaotic signal of Chua's circuit, and also better maintain the phase space structure and smoothness of the signal.

**Figure 8:**
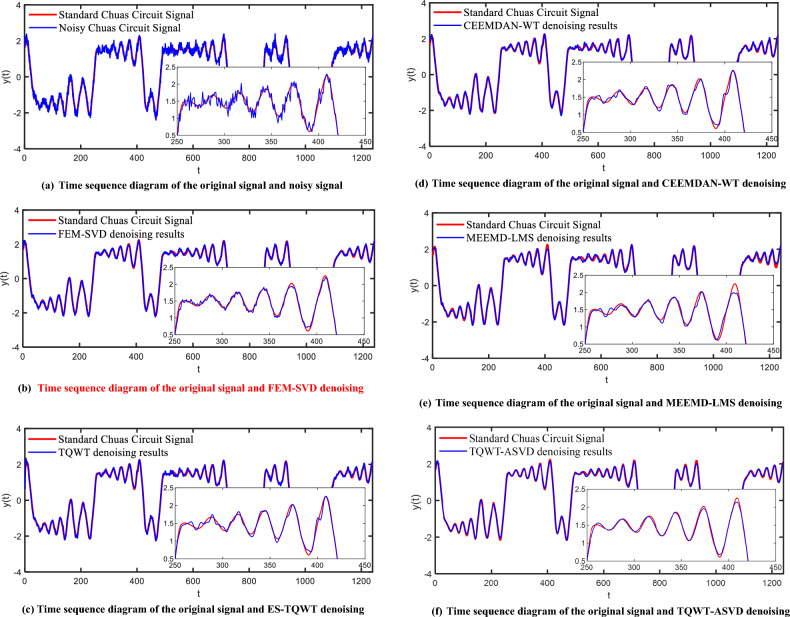
Comparison of signal timing graphs of noisy Chua's chaotic signal after FEM-SVD, TQWT, CEEMDAN-WT, MEEMD-LMS, and TQWT-ASVD methods.

The comparison between the five denoising methods and the original signal is shown in Fig. 8. Intercept the part of $t$ from 250 to 450 for local magnification (a small part of Fig. 8). By comparing the waveforms of the de-noising signal and the original Chua's circuit chaotic signal, it can be seen that although the five methods can remove the noise in the chaotic signal better, the FEM-SVD method has lost a lot of detailed information after de-noising, especially at the breakpoint, the signal amplitude after de-noising has a large deviation from the original signal amplitude. Although the amplitude of the TQWT, CEEMDAN-WT, and MEEMD-LMS methods is similar to the original signal, there are many noise disturbances near the breakpoint. The denoised signal in this paper is smoother and has less amplitude error than the original signal, and can better retain the abrupt information of the signal at the abrupt point of the original signal. By comparing the noise reduction waveforms of the five methods, it can be seen that the denoised signal in this method is smoother, flat, and closer to the original signal, and has a better noise reduction effect.

**Figure 9:**
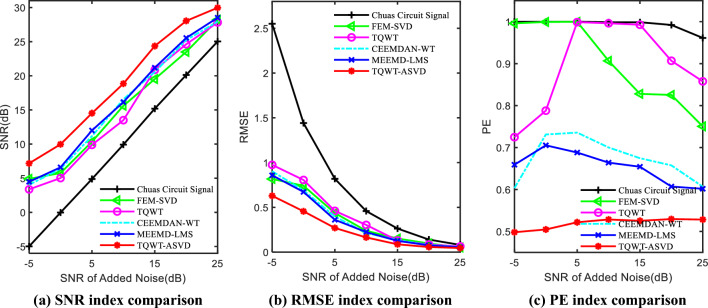
SNR, RMSE, and PE after noise reduction under different noise intensity.

To compare the noise reduction effects of the three methods at different noise intensities, Gaussian white noise with SNR of 5, 10, 15, 20, and 25 were added to Chua's circuit chaotic sequence respectively, and the SNR, RMSE, and PE of the three methods at different noise intensities were calculated. The larger the SNR, the smaller the RMSE and PE, the better the noise reduction effect. Considering the randomness of the added Gaussian white noise, 20 experiments were conducted for each noise intensity, and the average value was taken as the final experimental result. The detailed experimental results of the three methods under different noise intensities are shown in Table 1 and Fig. 9.

**Table 1:** SNR, RMSE, and PE indexes after noise reduction with different noise intensities.

Index	Noise (dB)	Observed signal	FEM-SVD	TQWT	CEEMDAN-WT	MEEMD-LMS	TQWT-ASVD
SNR	− 5	− 4.9867	4.9033	3.3684	4.0351	4.4773	7.1734
	0	− 0.0299	5.7536	5.0213	6.3739	6.5920	9.9678
	5	4.8822	10.3436	9.8792	11.1640	11.9920	14.5221
	10	9.9199	15.6098	13.5005	16.5561	16.1488	18.8470
	15	15.1918	19.5213	20.8698	20.5611	21.1821	24.3642
	20	20.1231	23.4926	24.6230	25.2248	25.5472	28.0388
	25	25.0310	28.2161	27.8189	27.9416	28.5083	29.9674
RMSE	− 5	2.5506	0.8168	0.9747	0.9027	0.8579	0.6290
	0	1.4415	0.7407	0.8058	0.6896	0.6725	0.4559
	5	0.8188	0.4366	0.4606	0.3973	0.3612	0.2699
	10	0.4585	0.2381	0.3036	0.2136	0.2238	0.1640
	15	0.2631	0.1518	0.1300	0.1347	0.1254	0.0869
	20	0.1400	0.0961	0.0844	0.0787	0.0758	0.0569
	25	0.0805	0.0517	0.0635	0.0576	0.0539	0.0456
PE	− 5	0.9997	0.9960	0.7247	0.6032	0.6590	0.4981
	0	0.9996	0.9993	0.7879	0.7311	0.7053	0.5047
	5	0.9999	0.9997	0.9989	0.7356	0.6881	0.5219
	10	0.9987	0.9066	0.9962	0.7002	0.6640	0.5285
	15	0.9987	0.6279	0.9927	0.6746	0.6542	0.5254
	20	0.9922	0.8254	0.9068	0.6575	0.6070	0.5295
	25	0.9613	0.7505	0.8577	0.6068	0.6017	0.5283

From Fig. 9a, when the noise intensity in the chaotic signal is relatively small (i.e. when the SNR is large), the SNR after denoising by the TQWT-ASVD method proposed in this paper is not significantly different from the SNR after denoising by the other four methods. For example, when the SNR = 25 dB, the SNR of the TQWT-ASVD method is only about 2 dB higher than that of the FEM-SVD, TQWT, CEEMDAN-WT, and MEEMD-LMS methods. However, as the noise intensity increases, the SNR improvement amplitude after noise reduction by the TQWT-ASVD method gradually increases. When SNR = − 5 dB, the SNR after noise reduction by the proposed method increases by about 2.98 dB on average compared with the other four methods.

From Fig. 9b, the denoised RMSE and SNR of the proposed method have a similar trend. When the SNR is large (such as SNR ∈ [15 dB, 25 dB]), although the TQWT-ASVD method has the smallest RMSE after noise reduction, compared with the other four methods, the reduction in RMSE is not significant. For example, when SNR = 25 dB, RMSE only decreases by about 0.02. But when the SNR of chaotic signals is small (such as SNR ∈ [− 5 dB, 15 dB]), the RMSE of the TQWT-ASVD method after noise reduction is significantly lower than that of the other four methods. When SNR = − 5 dB, the RMSE decreases by about 0.26 on average.

From Fig. 9c, chaotic signals with different SNR, the PE values of the signals denoised by both FEM-SVD and TQWT methods are relatively large and unstable, and the average PE value of FEM-SVD is about 0.8722, while the average PE value of TQWT method is about 0.8949. This shows that the denoising effect of FEM-SVD and TQWT methods is not stable, and the complexity of the denoised chaotic signal is high and irregular. Compared with the FEM-SVD and TQWT methods, E decreases significantly and is relatively stable after noise reduction by CEEMD-WT and MEEMD-LMS methods. The average PE value of the MEEMD-LMS method is about 0.6542, while the average PE value of the CEEMD-WT method is about 0.6727. The PE value of the MEEMD-LMS method is slightly lower than that of the CEEMD-WT method. This shows that the CEEMD-WT and MEEMD-LMS methods can effectively improve the denoising effect of chaotic signals, and the denoised signals are relatively regular. After noise reduction, the average PE value of the proposed method is about 0.5195, which is significantly lower than the average value of the CEEMD-WT and MEEMD-LMS methods. Moreover, the PE value of this method is stable at around 0.52, and there is no obvious fluctuation of PE value with the SNR decreases. This indicates that for chaotic signals with low SNR, the proposed method can still completely remove the noise from the signal, the signal complexity after denoising is relatively low and the data is relatively regular.

Based on the above experimental results, it can be concluded that under different noise intensities, the SNR of the signals denoised by the TQWT-ASVD method is the highest, while RMSE and PE are both the lowest. Especially when the SNR is low, the denoising effect of the TQWT-ASVD method is significantly better than the other four algorithms. The experimental results in Fig. 9 also demonstrate the effectiveness of the TQWT-ASVD method in denoising chaotic signals with low SNR.

As can be seen from Table 1, compared with the five methods for noise reduction, the SNR of the TQWT-ASVD method proposed in this paper is the largest, while RMSE and PE are the smallest. Compared with FEM-SVD, TQWT, CEEMDAN-WT, and MEEMD-LMS, SNR increased by 23.22%, 26.46%, 18.79%, and 16.11%, respectively. RMSE decreased by 32.53%,39.48%, 30.96%, and 27.94%, respectively. PE decreased by 40.44%, 41.96%, 22.78%, and 20.59%, respectively.

In Table 1, for 0 dB chaotic signals with low SNR, compared with FEM-SVD, TQWT, CEEMDAN-WT, and MEEMD-LMS methods, the SNR after noise reduction of the proposed method is increased by 70.78%, RMSE is reduced by 29.17%, and PE is reduced by 33.2%.

For − 5 dB chaotic signals with low SNR, compared with FEM-SVD, TQWT, CEEMDAN-WT, and MEEMD-LMS methods, the SNR after noise reduction of the proposed method is increased by 67.94%, RMSE is reduced by 37.3%, and PE is reduced by 37.37%.

From the denoising results of the newly added low SNR chaotic signal, it can be seen that the proposed method can also obtain better denoising results for the low SNR chaotic signal. Moreover, it can be seen from Table 1 that when the added noise SNR is within the range of 25 to − 5, the advantages of this method become increasingly apparent as the noise SNR ratio decreases.

In conclusion, the noise reduction method based on TQWT-ASVD proposed in this paper can obtain better noise reduction effects and higher reliability when de-noising chaotic signals. While effectively removing noise and maintaining the overall smoothness of the signal system, the signal details are well preserved.

### Noise reduction of ship underwater radiation signal

**Figure 10:**
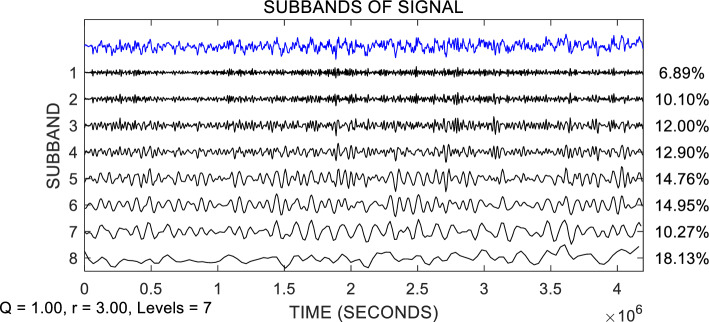
TQWT decomposition results of underwater radiation signals from cargo ships.

Ship underwater radiation signal has nonlinear, non-stationary, and chaotic characteristics, and its research has attracted wide attention. To verify the noise reduction performance of the proposed method on actual ship signals, cargo ship radiated noise was selected as the experimental data. The selection of measured data comes from the National Park Service. Vessel Sounds in the Sounds Recorded in Glacier Bay Plate (https://www.nps.gov/glba/learn/nature/soundclips.htm). The recordings of vessels were made at various distances from the hydrophone, and under a variety of sea conditions, but demonstrate that different types of vessels can be distinguished from one another. FEM-SVD, TQWT, CEEMDAN-WT, MEEMD-LMS, and the proposed method (TQWT-ASVD) were used to denoise the underwater radiation signals of cargo ships. In the TQWT method and the TQWT-ASVD method proposed in this paper, the Q-factor of TQWT is set as $Q$ = 1, the redundancy factor as $r$ = 3, and the number of decomposition layers as $J$ = 7. A total of 8 subbands are obtained, and the signal and energy proportion of each subband are shown in Fig. 10. The first line in the figure is the observed underwater radiated noise of the cargo ship, and the second line to the ninth line is the TQWT decomposition result. Among them, subband 8 can be considered as the signal subband, and subband 1 can be considered as the noise subband, but it is difficult to determine whether subband 2 to subband 7 is the noise part or the signal part, and the noise part needs to be removed by SVD processing.

**Figure 11:**
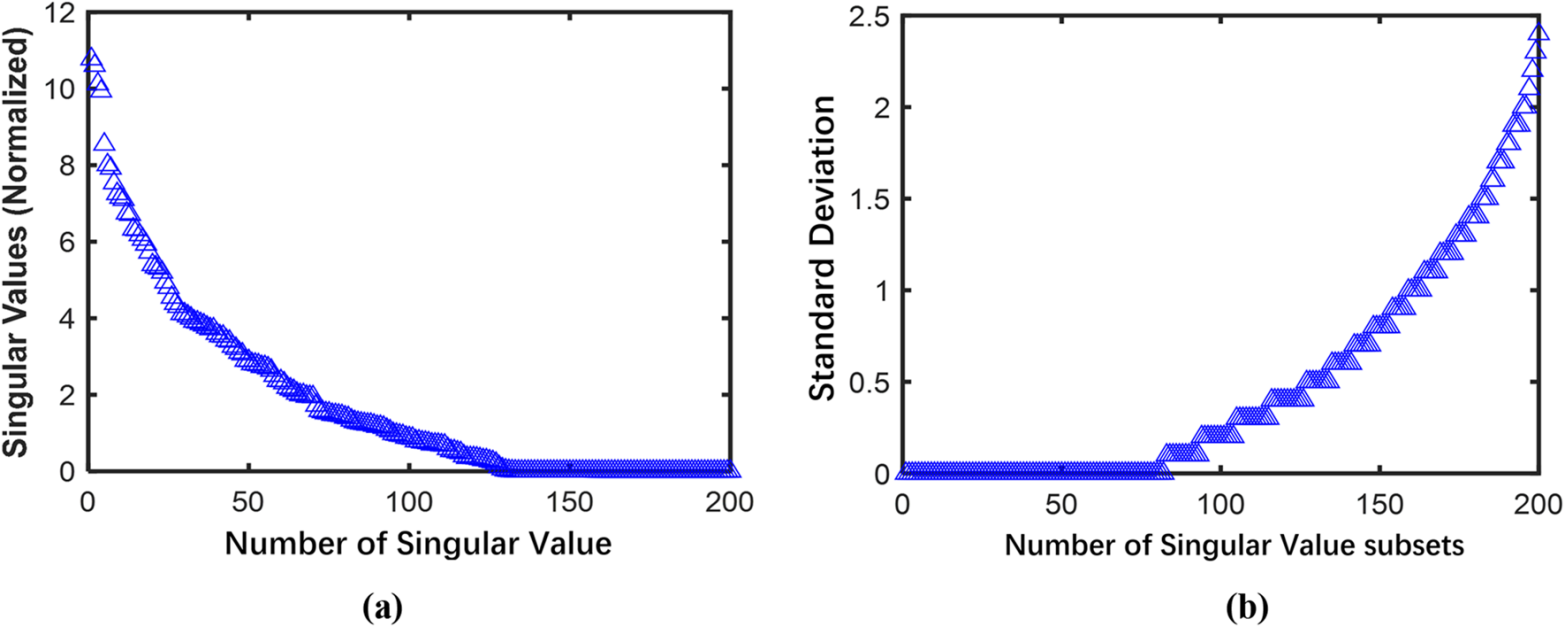
SVD order determination results: (**a**) Calculation of singular value normalization; (**b**) Calculation of deviation of singular value subset.

In the proposed method, the noise subband and signal subband after TQWT decomposition are selected according to formulas (16) and (17), and the noise subband is denoised using the SVD method of singular value subset order determination. The order selection diagram of the noise subband 1 when using singular value subset order determination is shown in Fig. 11. Compared with figure (a), figure (b) can see the boundary point of singular value more clearly. Hence the same SVD method is used to process subbands 2–7. The signal is reconstructed by noise subband and signal subband after noise reduction, and the reconstructed signal is denoised again by ASVD.

**Figure 12:**
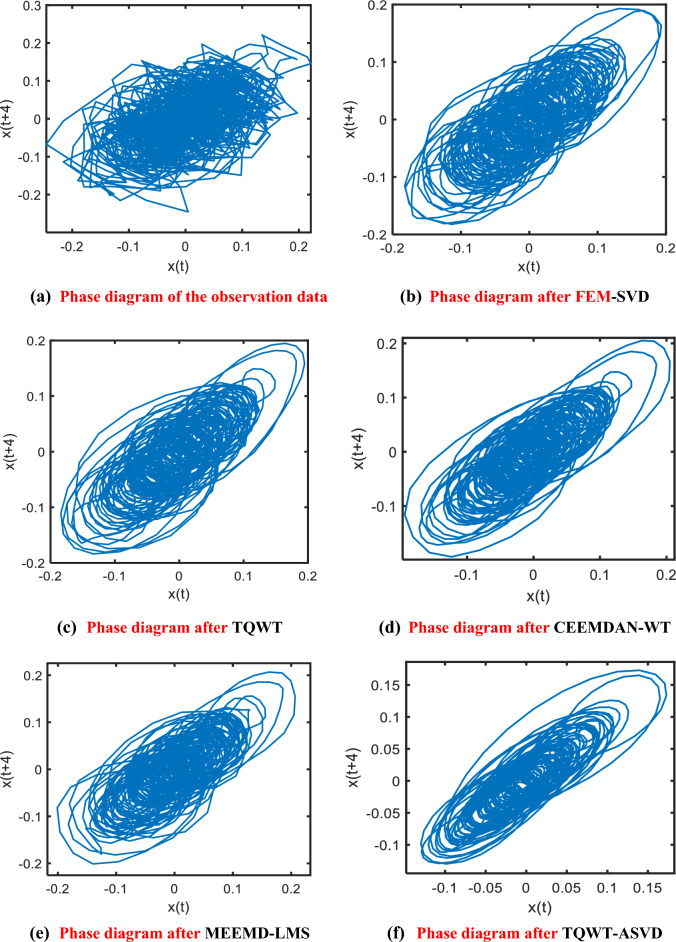
Phase-space comparison of noise reduction effects of underwater radiation signals of cargo ships: (**a**) Observation data; (**b**) FEM-SVD; (**c**) TQWT; (**d**) CEEMDAN-WT; (**e**) MEEMD-LMS; (**f**) TQWT-ASVD.

The original phase space trajectory diagram of the underwater radiation signal sequence of the cargo ship and the phase space trajectory after noise reduction by the five methods are shown in Fig. 12. By comparing Fig. 12a, it can be seen that due to noise interference in the process of data acquisition, the phase space trajectory of the underwater radiation signal sequence of the original cargo ship is very messy. In comparison Fig. 12b and e, after noise reduction by the FEM-SVD and MEEMD-LMS methods, the noise in the signal is removed to a certain extent., and the phase space manifold structure is much clearer, but because the noise is not completely removed, the phase space manifold structure curve of the sequence after noise reduction is not smooth, and even some curves overlap together and are difficult to distinguish.

It can be seen from Fig. 12c and d that after the sequence is denoised by TQWT and CEEMDAN-WT, the noise is better suppressed and the phase space trajectory becomes smoother, but the overall manifold structure is still not regular enough, some manifold structure curves are interlaced, and the geometric structure of the chaotic attractor is not clear enough. It can be seen from Fig. 12f that the two-dimensional phase space trajectory after noise reduction in this method is more smooth and regular, the manifold curves in phase space are basically separated from each other, and the geometric structure of the chaotic attractor of the original signal can be more clearly displayed. Therefore, the phase space trajectory after noise reduction of this method is also better than the other two methods.

**Figure 13:**
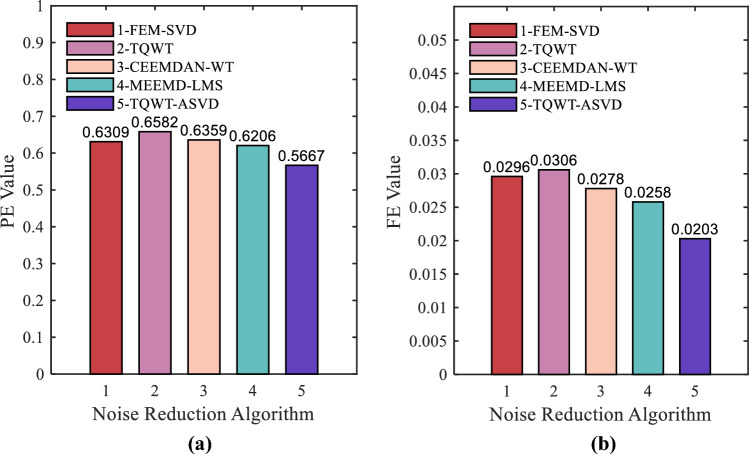
Analysis of signal denoised by different algorithms: (**a**) The PE of denoising results; (**b**) The FE of denoising results (1: FEM-SVD; 2: TQWT; 3: CEEMDAN-WT; 4: MEEMD-LMS; 5: TQWT-ASVD).

The five denoising methods can effectively remove interference noise from the original sequence. The normalized arrangement entropy values after denoising by the three methods are shown in Fig. 13. As can be seen from Fig. 13, the PE value and FE of the signal after noise reduction by the proposed method are significantly lower than those of the FEM-SVD, TQWT, CEEMDAN-WT, and MEEMD-LMS methods. PE value decreased by 13.91%, 10.18%, 10.88%, and 8.68%, and FE value decreased by 33.66%, 31.42%, 26.98%, and 21.32% respectively. This shows that the second denoising method proposed in this paper removes most of the noise in the sequence, and on the premise of ensuring the denoising effect, the denoised signal has better smoothness, stronger stability, and a more regular geometric structure.

**Figure 14:**
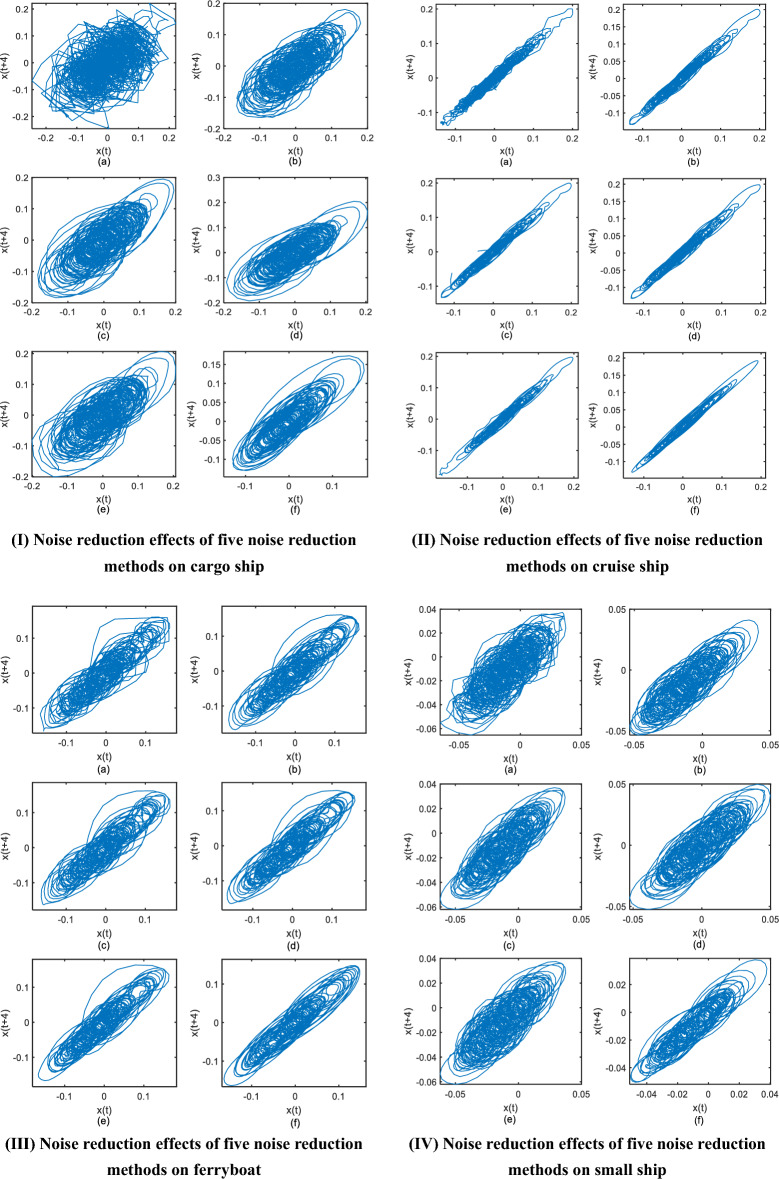
Phase diagrams after by noise reduction by five methods on four types ship-radiated signal, in the four subgraphs I, II, III, IV, (**a**) Original phase diagram of ship-radiated signal; (**b**) Phase diagrams after FEM-SVD denoising; (**c**) Phase diagrams after TQWT denoising; (**d**) Phase diagrams after CEEMDAN-WT denoising; (**e**) Phase diagram after MEEMD-WT denoising; (**f**) Phase diagram after TQWT-ASVD denoising;

It can be seen from Fig. 14 that the phase diagrams of the four types of ship radiated noise before denoising are chaotic, and the fractal characteristics of their attractors are difficult to observe. Compared with other noise reduction methods, the geometric structure of the phase diagram after noise reduction with TQWT-ASVD is more regular and smooth, the four kinds of ship radiated noise after noise reduction have been well eliminated.

From Table 2, the noise reduction effect of the TQWT-ASVD method for the measured chaotic signal is generally better than the FEM-SVD, TQWT, CEEMDAN-WT, and MEEMD-LMS methods. The four underwater radiated signal sequences after noise reduction by this method have smaller normalized permutation entropy and fuzzy entropy, which reflects the effectiveness of this method.

**Table 2:** Comparison of PE and FE indicators for noise reduction effects of five methods.

Index	Type	Observed signal	ES-TQWT	FEM-SVD	CEEMDAN-WT	MEEMD-LMS	TQWT-ASVD
PE	Small ship	0.7860	0.6455	0.6354	0.6570	0.6659	0.6277
	Cruise ship	0.8626	0.5567	0.5620	0.5455	0.5404	0.4621
	Ferryboat	0.7363	0.6582	0.6309	0.6359	0.6206	0.5667
	Cargo ship	0.8374	0.6617	0.6986	0.6447	0.6595	0.5815
FE	Small ship	0.0222	0.0155	0.0146	0.0158	0.0179	0.0074
	Cruise ship	0.0044	0.0030	0.0030	0.0028	0.0029	0.0019
	Ferryboat	0.0328	0.0306	0.0296	0.0278	0.0258	0.0203
	Cargo ship	0.1323	0.0601	0.0602	0.0514	0.0646	0.0272

According to the analysis results in Fig. 14 and Table 2, the TQWT-ASVD method proposed in this paper is generally superior to SVD, TQWT, CEEMDAN-WT, and MEEMD-LMS methods in noise reduction of measured chaotic signals. Using a qualitative and quantitative comparison of the ship’s radiated noise, the results show that TQWT-ASVD can effectively applied in ocean environmental noise radiated noise of the ship.

## Conclusion

In this paper, a chaotic signal denoising method combining TQWT and adaptive singular value decomposition (ASVD) is proposed. In the theoretical statement and experimental analysis, the following conclusions are obtained:

1. Different from discriminating signal and noise subbands based on correlation coefficient, this method adaptively discriminates noise subbands and signal subbands of chaotic signals according to energy threshold based on the different energy ratios of signal components and noise components after TQWT decomposition. The results of the TQWT decomposition of model unknown signals provide a more advantageous method for distinguishing different types of subbands.
2. The subband judged to be dominated by noise still contains some weak real signals. The SVD method with the variance of singular value subset is used to filter the noise subband, which preserves more detailed signals, improves the noise reduction effect, and realizes the initial noise reduction of chaotic signals. The subband determined to be dominated by the real signal still contains some weak noise signals. TQWT reconstruction is carried out on the filtered noise subband and signal subband, and the improved SVD is used to carry out secondary noise reduction on the reconstructed chaotic signal, further filtering out the noise in the chaotic sequence, keeping the geometric structure of the chaotic signal clear and making the denoised signal smoother.
3. Noise reduction experiments were conducted on Chua's chaotic sequence known to the model and underwater radiated noise signals unknown to the model. The experimental results show that The main and objective evaluation of the proposed method is superior to the TQWT method, SVD method, CEEMDAN-WT method, and MEEMD-LMS method, which proves the effectiveness of the proposed method.
4. The advantage of the TQWT method is that it can flexibly adjust parameters to achieve the best decomposition effect, and it can be applied to the denoising of more complex observed chaotic noise such as chaotic circuits, ECG signals, and EMG signals. In the follow-up research, the selection of optimal parameters for TQWT decomposition of the observed chaotic signals and the definition of the mutation point location of the SVD subset can be discussed in more detail, which provides a basis for the analysis of chaotic systems.

## Data Availability

The selection of experimental data comes from the National Park Service. Vessel Sounds in the Sounds Recorded in Glacier Bay Plate (https://www.nps.gov/glba/learn/nature/soundclips.htm). All data generated or analyzed during this study are included in this published article.
